# Cisplatin Induces Resistance by Triggering Differentiation of Testicular Embryonal Carcinoma Cells

**DOI:** 10.1371/journal.pone.0087444

**Published:** 2014-01-27

**Authors:** Paolo B. Abada, Stephen B. Howell

**Affiliations:** Department of Medicine and the Moores UCSD Cancer Center, University of California San Diego, La Jolla, California, United States of America; The Institute of Cancer Research, London, United Kingdom

## Abstract

Although testicular germ cell tumors are generally quite responsive to treatment with cisplatin, a small fraction of them acquire resistance during therapy. Even when cisplatin treatment is successful the patient is often left with a residual teratoma at the site of the primary tumor suggesting that cisplatin may trigger differentiation in some tumors. Using the human embryonal carcinoma cell line NTera2/D1, we confirmed that exposure to the differentiating agent retinoic acid produced a reduction in pluripotency markers NANOG and POU5F1 (Oct3/4) and an acute concentration-dependent increase in resistance to both cisplatin and paclitaxel that reached as high as 18-fold for cisplatin and 61-fold for paclitaxel within four days. A two day exposure to cisplatin also produced a concentration-dependent decrease in the expression of the NANOG and POU5F1 and increased expression of three markers whose levels increase with differentiation including Nestin, SCG10 and Fibronectin. In parallel, exposure to cisplatin induced up to 6.2-fold resistance to itself and 104-fold resistance to paclitaxel. Paclitaxel did not induce differentiation or resistance to either itself or cisplatin. Neither retinoic acid nor cisplatin induced resistance in cervical or prostate cancer cell lines or other germ cell tumor lines in which they failed to alter the expression of NANOG and POU5F1. Forced expression of NANOG prevented the induction of resistance to cisplatin by retinoic acid. We conclude that cisplatin can acutely induce resistance to itself and paclitaxel by triggering a differentiation response in pluripotent germ cell tumor cells.

## Introduction

In contrast to most other cancers, testicular germ cell tumors (GCTs) have a very high cure rate of >90% even when the disease is widely metastatic at presentation. The reason appears to be their exquisite sensitivity to platinum (Pt) drug-containing chemotherapy that is the backbone of current treatment regimens. Although this observation was made decades ago, the reason why they are initially so sensitive to the Pt drugs remains unknown. Attempts to identify mechanisms underlying the initial sensitivity of GCTs to the Pt drugs have included studies of: 1) drug accumulation; 2) drug detoxification; 3) DNA repair; and, 4) apoptotic mechanisms [1], [2]. However, results have been conflicting and there remains uncertainty as to what cellular pathways are the most important with regard to the initial sensitivity of the tumor or the emergence of resistance during treatment [3]–[6].

Despite their initial sensitivity, there is a significant fraction of patients whose tumors acquire resistance during therapy. There are a variety of subtypes of testicular GCTs that are classified on the basis of their apparent degree of differentiation. Testicular GCTs are broadly classified as seminomas or non-seminomatous. Seminomas are of only one histologic type and are considered to be relatively undifferentiated. In contrast, non-seminomatous GCTs are classically divided into four histologic types including embyronal, yolk-sac, choriocarcinoma, and teratomatous. Among these, embryonal carcinoma is considered to be the most undifferentiated type of GCT [1]. Seminomas are associated with a better clinical prognosis and are highly sensitive to chemotherapy. In contrast, non-seminomatous GCTs have a worse prognosis and in general can be much more resistant to systemic therapy and so are treated more aggressively. In some cases resistance evolves in the absence of any change in histology, but in others resistance is associated with the emergence of more differentiated teratomatous elements. This latter observation suggests that initial sensitivity and acquired resistance are related to the state of differentiation. GCTs are believed to arise from embyronal germ cells which are already intrinsically sensitive to DNA damaging agents and this partly explain unusual initial sensitivity to chemotherapy [1], [7], [8]. Prior studies have reported that GCTs previously treated with either cisplatin (cDDP) or carboplatin have mRNA expression profiles that are similar to the most differentiated types of GCTs suggesting that drug treatment may induce differentiation [9]. This concept is supported by the clinical observation that tumor masses that persist following Pt drug therapy usually have a differentiated teratomatous histology.

We report here that cisplatin is capable of inducing changes in embryonal carcinoma cells consistent with induction of differentiation, and that this results in rapid appearance of resistance to both cDDP and paclitaxel. Treatment of cells with cDDP decreased expression of transcription factors NANOG and POU5F1 that maintain the undifferentiated state. It also led to increases in nestin, Scg10 and fibronectin which are markers associated with differentiation, and increased resistance to cDDP and paclitaxel in a manner similar to the effect of the differentiating agent retinoic acid (RA). Cell lines established from GCT and other types of tumors in which RA and cDDP failed to down-regulate NANOG and POU5F1 failed to acquire acute drug resistance. Pretreatment with paclitaxel was unable to induce a similar effect. Finally, over-expression of NANOG abrogated the ability of both RA and cDDP to induce resistance to cDDP demonstrating linkage between the ability to trigger differentiation and the induction of drug resistance.

## Materials and Methods

### Drugs and Reagents

A commercial formulation of cDDP was obtained from the Moores Cancer Center pharmacy. Paclitaxel was a gift from the San Diego Veterans Affairs Infusion Center Pharmacy. Retinoic acid, puromycin, and blasticidin were obtained from Sigma (Sigma, St. Louis, MO). Drugs were diluted to the desired concentrations in RPMI or DMEM medium (Thermo Scientific; Logan, UT). The Detergent Compatible Protein kit was purchased from BioRad (Hercules, CA) and crystal violet was obtained from Sigma-Aldrich (MP Biomedicals; Solon, OH).

### Cell Types, Culture, and Molecular Engineering

NTera2/D1 (NT2-D1) cells [10] (obtained from Dr. Nazneed Dewji, University of California, San Diego) were grown in DMEM medium supplemented with 10% fetal calf serum, 1 mM sodium pyruvate, 2 mM glutamine, and penicillin-streptomycin. NT2-D1 cells are believed to represent embyronal carcinoma and were derived from a lung metastases of a patient with metastatic testicular carcinoma [8]. Human cervical carcinoma 2008 [11], prostate cells PC3 [12] and DU145 [13], GCT27 [14] and SuSa cells [15] (latter two lines obtained from Dr. John Masters, University of California, Los Angeles) were grown in RPMI medium supplemented with 10% fetal calf serum, sodium pyruvate, glutamine, and penicillin-streptomycin. Although the 2008 cell line was originally described as being isolated from a patient with ovarian cancer [11], genetic testing has recently shown that this line is identical to the ME-180 cervical carcinoma cell line. For pretreatment with various compounds, the specified drug was added to the complete medium at the indicated concentration for the duration specified.

The NT2-EV and NT2-NANOG cells were constructed by infecting NT2-D1 cells with a retrovirus expressing either the empty pCX4 vector or human *NANOG* using the pCL-Ampho system with pCX4bsr retrovirus vectors kindly provided by Dr. Steve Dowdy (University of California, San Diego) [16]. Following transduction, cells were selected using blasticidin at 5 µM for 7–10 days before use, and maintained in 5 µM thereafter.

### Western Blot Analysis

Whole-cell lysates were dissolved in lysis buffer [150 mmol/L NaCl, 5 mmol/L EDTA, 1% Triton X-100, and 10 mmol/L Tris (pH 7.4)] with protease inhibitor (Roche; Mannheim, Germany) and subjected to electrophoresis on 4% to 15% Tris-glycine gels using 50–80 µg of protein per lane. Protein levels were first determined by the DC protein Assay (Bio-Rad; Hercules, CA). A Bio-Rad Trans-Blot system was used to transfer the proteins to Immobilon-P FL membranes (Millipore; Bedford, MA). Blots were incubated overnight at 4°C in 5% dry nonfat milk in TBS (150 mmol/L NaCl, 300 mmol/L KCl, 10 mmol/L Tris (pH 7.4), 0.01% Tween 20). Blots were incubated for 16 h at 4°C with anti-POU5F1 antibody at a dilution of 1∶500 (Santa Cruz; Santa Cruz, CA), anti-NANOG antibody at a 1∶100 dilution (Santa Cruz; Santa Cruz, CA), or antibody to β-actin (Santa Cruz; Santa Cruz, CA). A fluorescently labeled secondary antibody (Li-Cor; Lincoln, NE) was dissolved in 5% milk in the TBS-T buffer and incubated with the blot for 2 h at room temperature. After three 10 min washes, blots probed with fluorescently labeled antibody were imaged using an Odyssey Infrared Imager (Li-Cor; Lincoln, NE).

### Cell survival assay

Cell survival following exposure to increasing concentrations of drugs was assayed using a crystal violet assay system. Three to four thousand cells were seeded into each well of a 96-well tissue culture plate. Cells were incubated overnight at 37°C in 5% CO_2_ and then exposed to varying drug concentrations in 200 µL complete medium. Cells were allowed to grow for 4 days following the addition of drug after which the medium was removed and washed once with room temperature PBS. After washing, the cells were then fixed and stained with 0.5% w/v crystal violet in 20% methanol for 30 minutes, washed four times with distilled water, and allowed to dry. The crystal violet stain in each well was then re-dissolved in 100 µL of Sorensen's buffer with shaking for 15 to 30 minutes, and the absorbance of each well at 595 nm was recorded using a Versamax Tunable Microplate Reader (Molecular Devices; Sunnyvale, CA). Results are plotted as drug concentration versus log10 cell survival. All experiments were repeated at least 3 times using 3 cultures for each drug concentration.

### qRT-PCR

mRNA levels for genes described were measured using qRT-PCR. cDNA was generated from mRNA isolated using TRIzol (Invitrogen; Carlsbad, CA). Purified mRNA was converted to cDNA using iScript Reverse Transcription Supermix (Bio-rad) and qRT-PCR was performed on an MyIQ qPCR machine (Bio-Rad Laboratories; Hercules, CA). Reactions were prepared using iTaQ SYBR Green Supermix (Bio-Rad Laboratories; Hercules, CA) according to the manufacturer's recommendations. Samples were prepared in quadruplicate with at least three independent sample sets being analyzed. Analysis was done using the Bio-Rad iQ5 system software.

### Statistics

All two-group comparisons utilized Student's *t*-test with the assumption of unequal variance. Data are presented as mean ±SEM.

## Results

### Retinoic Acid-Induced Differentiation Renders NT2-D1 Cells Resistant to cDDP and Paclitaxel

The NTera2/D1 (NT2-D1) cell line is believed to represent the embryonic subtype of testicular carcinoma and was isolated from a patient with metastatic testicular carcinoma [8]. Clinically this subtype is relatively cDDP sensitive, and this was confirmed by the finding a cDDP IC_50_ of 0.23 µM (Table 1) which is 4–10 times lower than, for example, ovarian cancer cell lines, and is consistent with previous observations [17], [18].

**Table 1:** IC_50_ values for cDDP and paclitaxel in NT2-D1 cells pretreated with retinoic acid.

	cDDP IC_50_ µM	Fold increase in IC_50_ induced by pretreatment	P Value	Paclitaxel IC_50_ µM	Fold increase in IC_50_ induced by pretreatment	P Value
NT2-D1 No pretreatment	0.23±0.01	1	-	0.0018±0.00001	1	-
NT2-D1 pretreated with 0.01 µM RA	0.40±0.04	1.7	<0.01	0.0018±0.00004	1	NS
NT2-D1 pretreated with 0.1 µM RA	3.05±0.61	13.3	<0.01	0.0026±0.00004	1.4	<0.001
NT2-D1 pretreated with 10 µM RA	4.28±0.28	18.6	<0.001	0.11±0.01	61	<0.01

**Figure 1:**
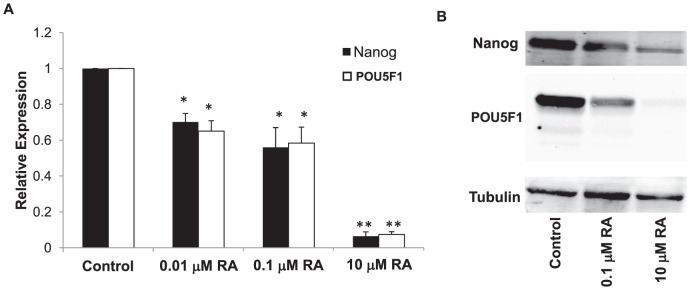
Retinoic acid induces differentiation of NT2-D1 embryonal carcinoma cells. Cells were exposed to RA for 4 days prior to examination of NANOG and POU5F1 levels. A) qRT-PCR analysis; B) western blot analysis. qRT-PCR data was normalized to GAPDH. Each bar in the histogram represents the mean of 3 independent experiments; vertical bars, ±SEM. * p<.05, ** p<0.001.

A characteristic of embryonal carcinomas that is also exhibited by NT2-D1 cells is their ability to differentiate and acquire features of more histologically mature cells, supporting the hypothesis that these cells contain a stem cell population. NT2-D1 cells have the capability to differentiate into non-embryonic tissues in response to RA, and this occurs primarily along a neuronal differentiation pathway [19]. We confirmed this observation by treating NT2-D1 cells with increasing concentrations of RA. As shown in Figure 1A, exposure to even low concentrations of RA for 4 days led to decreases in the mRNA levels of *NANOG* and *POU5F1*, both of which are markers of pluripotency. This was confirmed at the protein level by western blot analysis (Figure 1B). These decreases were apparent as soon as one day following addition of RA, even before any clear morphological changes were apparent, indicating that NANOG and POU5F1 are down-regulated early in the differentiation process (Supplementary Figure S1).

**Figure 2:**
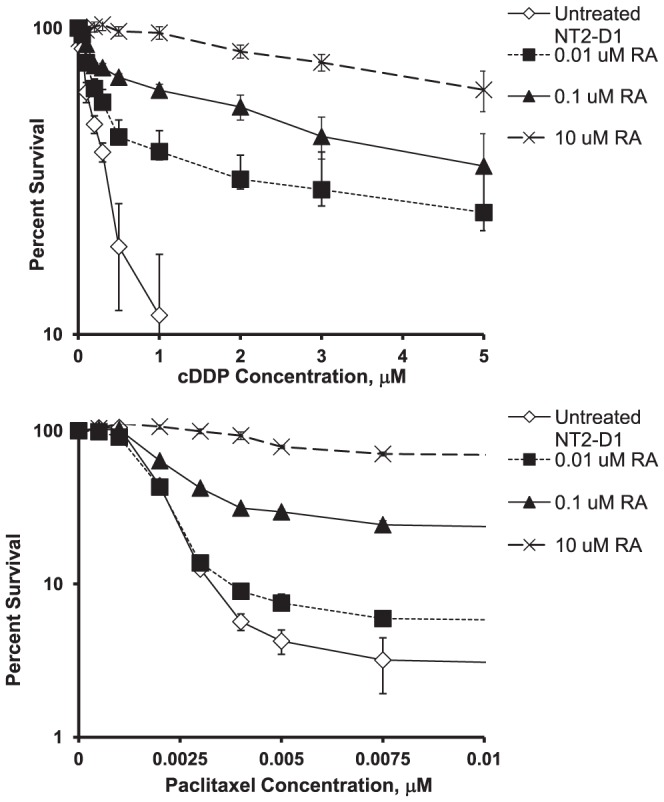
Effect of RA pretreatment of NT2-D1 cells on sensitivity to the growth inhibitory effect of cDDP and paclitaxel. NT2-D1 cells were exposed to different concentrations of RA for 4 days then replated and exposed to cDDP or paclitaxel continuously for 4 days. Survival was measured using a crystal violet staining assay. Each data point represents the mean of at least four independent experiments each performed with triplicate cultures. Vertical bars, ±SEM.

GCTs having more differentiated histologies than embryonal carcinoma have been reported to be more drug resistant both *in vitro* and in patients [20], [21]. Accompanying their differentiation, exposure of NT2-D1 cells to RA for 4 days was enough to induce a cDDP resistant phenotype. Concentrations of 0.1 or 1.0 µM RA had no discernible effect on growth rate, but clearly rendered the cells resistant to cDDP as shown by concentration-survival curves presented in Figure 2A. The parental cells had a cDDP IC_50_ of 0.23±0.01 µM; treatment with 0.01 µM RA for 4 days led to a 1.7-fold increase in cDDP IC_50_ to 0.40±0.04 µM. Treatment with 0.1 µM RA led to a 13.3-fold increase in cDDP IC_50_ to 3.05±0.61 µM, and 4 days of 10 µM RA treatment increased the IC_50_ 18.6-fold to 4.28±0.28 µM. The effect was clearly concentration-dependent, and although a slowing of cell proliferation might have played a role at the highest concentration of RA used, this could not explain the increase in cDDP resistance produced by the lower RA concentrations. These findings are consistent with those reported by Skotheim et al. [21].

Similar to its effect on sensitivity to cDDP, treatment with RA also rendered the NT2-D1 cells resistant to paclitaxel (Figure 2B). As for cDDP, this was most pronounced with the higher RA concentrations. Untreated NT2-D1 cells had a paclitaxel IC_50_ of 0.0018±0.00001 µM. Pretreatment with 0.01 µM RA for 4 days did not significantly increase resistance, but pretreatment with 0.1 µM RA increased it by 1.4-fold, and 10 µM RA increased it by 61-fold (Table 1). Since cDDP and paclitaxel have very different cellular pharmacology and intracellular targets, induction of differentiation by RA must affect several different drug resistance mechanisms.

### cDDP Triggers Differentiation of NT2-D1 Cells

**Figure 3:**
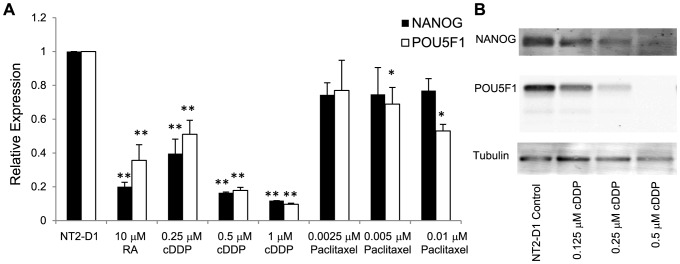
The effect of cDDP or paclitaxel on the expression of NANOG and POU5F1 in NT2-D1 cells. The expression of *NANOG* and *POU5F1* mRNA was measured in NT2-D1 cells following a 4 d exposure to increasing concentrations of RA, or 2 d exposures to cDDP or paclitaxel. A) qRT-PCR analysis; B) Western blot analysis. Each bar presents the results of measurements made in 3 independent experiments performed using triplicate cultures. Vertical bars, ±SEM. * p<.05, ** p<0.001.

**Figure 4:**
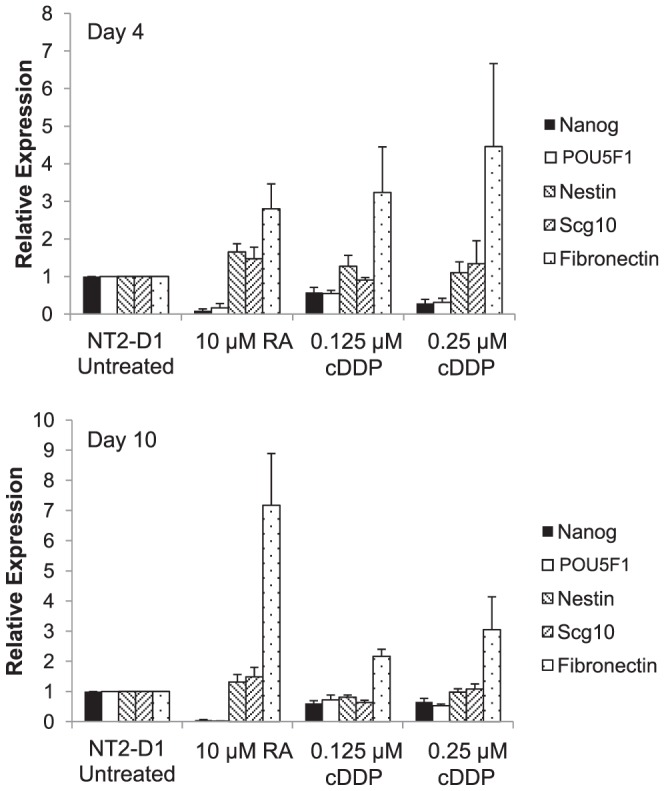
The effect of RA and cDDP on the expression of markers of differentiation in NT2-D1 cells. NT2-D1 cells were exposed to RA for 4 days or cDDP for 2 days. RNA was collected was collected before and at 4 and 10 days after initiation of RA or cDDP treatment and the levels *NANOG*, *POU5F1*, nestin, Scg10 and fibronectin mRNA were determined by qRT-PCR. Each bar presents the results of measurements made in 4 independent experiments performed using triplicate cultures. Vertical bars, ±SEM.

It has previously been reported that there are similarities in the gene expression profiles of the more differentiated histologic types of testicular cancer and the tumor masses that persist following cDDP or carboplatin treatment of initially undifferentiated tumors [21]. To determine whether cDDP triggers differentiation, NT2-D1 cells were exposed to increasing concentrations of cDDP for 48 h and the expression of *NANOG* and *POU5F1*was assessed by qRT-PCR 4 and 10 days after start of cDDP exposure. As shown in Figure 3, cDDP decreased the expression of NANOG and POU5F1 in a concentration-dependent manner at both the mRNA (Figure 3A) and protein level (Figure 3B) similar to what was observed following RA treatment. In contrast, over a similar range of cytotoxicity, paclitaxel failed to reduce the expression of either transcription factor. This was not due to a general inhibition of transcription since the changes in NANOG and POU5F1 expression were normalized to GAPDH expression. To provide additional evidence that cDDP was triggering a differentiation program and not just non-specifically decreasing transcription and translation, its effect on markers that are up-regulated during differentiation was compared to that of RA. As shown in Figure 4, after 4 days of exposure to RA the expression of nestin and SCG10 was significantly increased by 1.7-fold and 1.5-fold, respectively; this effect had begun to fade by day 10. cDDP produced a similar 1.3-fold increase in nestin and 1.3-fold increase in the expression of SCG10 at day 4 that also returned to baseline by day 10. Both of these markers are neuron-specific and may not reflect the effect of cDDP on the differentiation pathways fully available to testicular carcinoma cells. Fibronectin is another marker whose expression is increased during differentiation. As shown in Figure 4, both RA and cDDP produced a large increase in the expression of fibronectin at day 4. The effect was actually greater for cDDP than for RA at day 4, but whereas the effect of RA continued to increase by day 10, that of cDDP had begun to fade. The differences between RA- and cDDP- induced expression of differentiation markers suggests that the differentiation program activated by cDDP may differ from that of RA, possibly along a non-neuronal lineage as has been reported for embryonal carcinoma cells following treatment with other agents such as bone morphogenic proteins [22], [23].

### cDDP-Induced Differentiation is Accompanied by Drug Resistance

**Figure 5:**
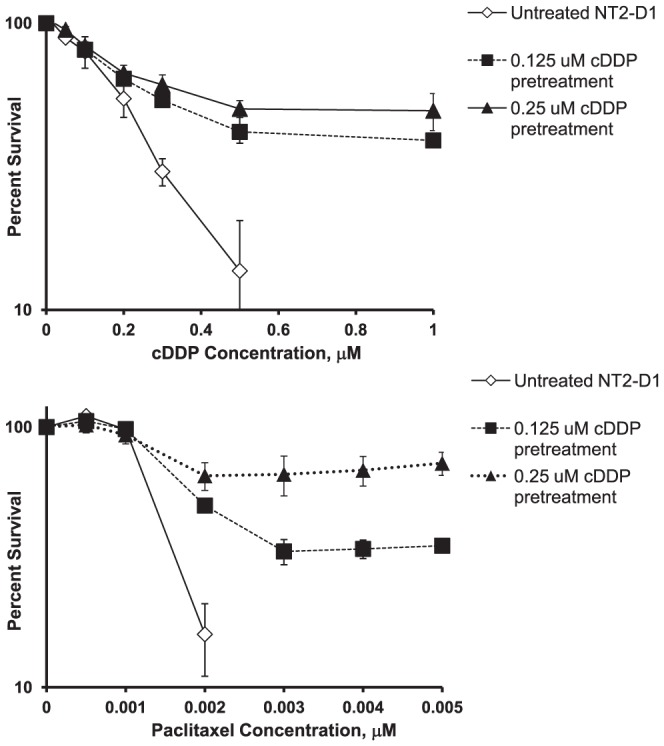
Effect of cDDP pretreatment of NT2-D1 cells on sensitivity to cDDP and paclitaxel. NT2-D1 cells were exposed to various concentrations of cDDP for 2 days then cultured in drug free media for 4 days following which they were replated and exposed continuously to cDDP or paclitaxel to generate concentration-survival curves. Each data point presents the mean of at least 4 independent experiments each performed with triplicate cultures. Vertical bars, ±SEM.

**Figure 6:**
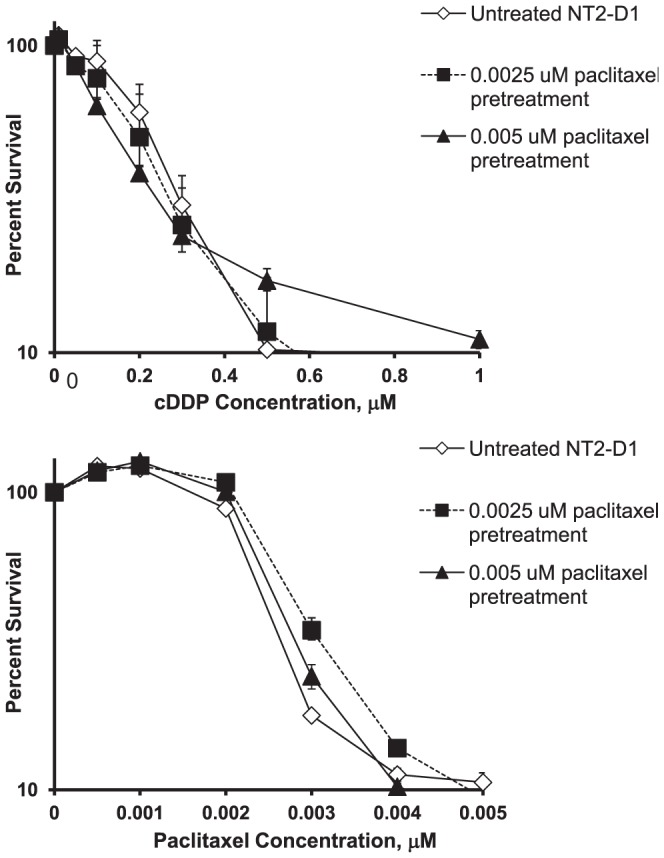
Paclitaxel pretreatment is unable to induce subsequent cDDP or paclitaxel resistance. NT2-D1 cells were exposed to various concentrations of paclitaxel for 2 days then cultured in drug free media for 4 days following which they were replated and exposed continuously to cDDP or paclitaxel to generate concentration-survival curves. Each data point presents the mean of 3 independent experiments each performed with triplicate cultures. Vertical bars, ±SEM.

If cDDP is inducing differentiation in NT2-D1 cells in a manner analogous to RA, then cDDP itself should induce a cDDP- and paclitaxel-resistant phenotype. NT2-D1 cells were treated with 0.125 or 0.25 µM cDDP for 48 h, the drug was then removed and the cells allowed to grow in drug-free media for 96 h following which they were re-plated and tested for sensitivity to cDDP and paclitaxel in a cytotoxicity assay. In previous studies using ovarian carcinoma cells, pre-treatment with cDDP failed to demonstrate any change in cDDP sensitivity under these experimental conditions (Howell, S.B., unpublished observations). As shown in Figure 5, and presented in Table 2, untreated NT2-D1 cells had a cDDP IC_50_ of 0.19±0.02 µM. A 48 h pretreatment with 0.125 µM cDDP resulted in a significant increase in the cDDP IC_50_ by 2.1-fold to 0.39±0.05 µM. Pretreatment with 0.25 µM cDDP increased the IC_50_ by 6.2-fold to 1.17±0.40 µM. To determine whether the resistance-inducing effect of cDDP was specific, its ability to induce resistance to paclitaxel was tested in parallel. Similar to the effect of RA, pretreatment of NT2-D1 cells with cDDP increased resistance to paclitaxel in a concentration-dependent manner (Figure 5 and Table 2). Conversely, pretreatment of cells with equivalently cytotoxic concentrations of paclitaxel (0.0025 or 0.005 µM) for 48 h did not result in a similar induction of resistance to cDDP or paclitaxel. As shown in Figure 6, the concentration-survival curves for untreated or paclitaxel-pretreated cells were almost superimposable. These results indicate that, like RA, cDDP induced a multidrug resistant phenotype.

**Table 2:** IC_50_ for cDDP and paclitaxel in various cell types pretreated with cDDP.

	cDDP IC_50_ µM	Fold Increase in IC_50_	p Value	Paclitaxel IC_50_ µM	Fold increase in IC_50_	p Value
NT2-D1 untreated	0.19±0.02	-		0.0014±0.00005	-	
NT2-D1 0.125 µM cDDP pretreated	0.39±0.05	2.1	<0.001	0.0020±0.00004	1.4	<0.001
NT2-D1 0.25 µM cDDP pretreated	1.17±0.41	6.2	<0.05	0.146±0.056	104	<0.05
2008 untreated	0.59±0.03	-				
2008 0.9 µM cDDP pretreated	0.42±0.05	0.71	<0.001			
PC3 untreated	2.0±0.5	-				
PC3 0.5 µM cDDP pretreated	1.7±0.4	0.85	NS			
DU145 untreated	1.37±0.04	-				
DU145 1.3 µM cDDP pretreated	1.23±0.05	0.90	<0.05			

### The Ability to Differentiate is a Prerequisite for Development of Resistance to cDDP

We were curious as to whether cDDP could induce changes in sensitivity to itself in cell lines derived from other types of cancer and chose cervical and prostate cancer lines for comparison. Human cervical carcinoma 2008 cells, and PC3 and DU145 cells derived from prostate cancers, were tested for the ability of equally cytotoxic concentrations of cDDP to induce cDDP resistance. As shown by the data presented in Table 2, unlike the observations in NT2-D1 cells, pretreatment of the cervical or prostate cancer cells with cDDP did not induce resistance to a subsequent exposure to cDDP and in some cases led to a small but statistically significant degree of sensitization.

**Figure 7:**
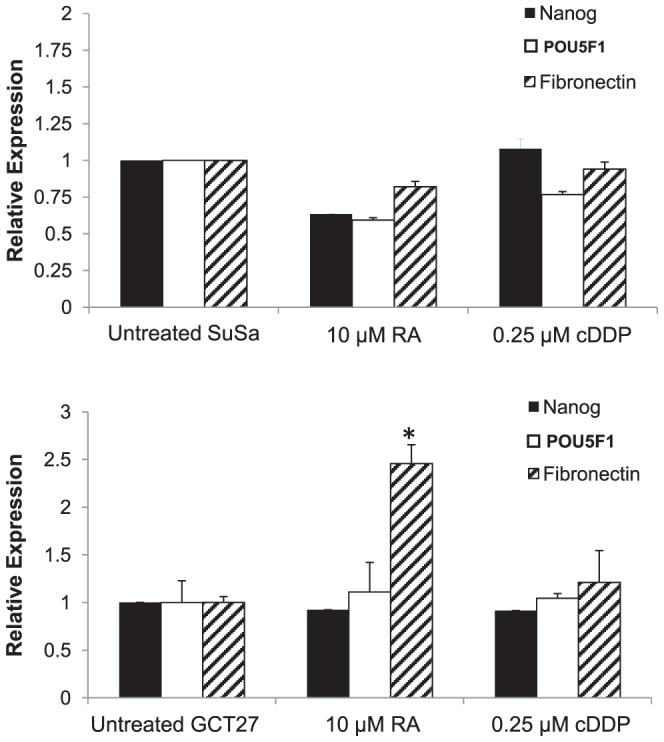
The effect of RA or cDDP on the expression of NANOG, POU5F1 and fibronectin in SuSa and GCT27 cells. SuSa and GCT27 cells were exposed to RA for 4 days or equitoxic concentrations of cDDP for 2 days. RNA was collected 4 days after initiation of treatment and the mRNA level of *NANOG*, *POU5F1* and fibronectin was quantified by qRT-PCR. Each bar presents the results of measurements made in 3 independent experiments performed using triplicate cultures. Vertical bars, ±SEM. *p<0.05.

To further explore the ability of cDDP to acutely induce resistance to itself, the effect was tested in the non-differentiating GCT lines GCT27 and SuSa. These lines do not normally differentiate in response to RA in culture and, if the ability to differentiate is essential to the acute induction of cDDP resistance, one would expect that these cell lines would not exhibit an acute change in cDDP sensitivity. Consistent with this hypothesis, neither RA or cDDP was able to induce a change in cDDP sensitivity in the SuSa line (Table 3). As shown in Figure 7A, SuSa cells also did not demonstrate any decrease in the mRNA for *NANOG* or *POU5F1*, or a significant increase in fibronectin, in response to either RA or cDDP. This provides further verification of the linkage between the ability of cDDP to induce differentiation and resistance.

**Table 3:** IC_50_ for cDDP in GCT lines pretreated with retinoic acid or cDDP.

	cDDP IC_50_ µM	Fold Increase	p Value
SuSa untreated	0.27±0.01	-	
SuSa 10 µM RA pretreated	0.28±0.05	1.0	NS
SuSa 0.25 µ cDDP pretreated	0.24±0.02	0.93	NS
GCT27 untreated	1.13±0.17	-	
GCT27 10 µM RA pretreated	1.66±0.20	1.5	<0.001
GCT27 0.5 µM cDDP pretreated	1.32±0.12	1.2	NS

In contrast to the Susa cells, the GCT27 cells showed a small response to RA. Pretreatment of GCT27 cells with RA was able to induce a relatively low but statistically significant 1.5-fold increase in cDDP resistance. However, pre-treatment with cDDP was not able to induce significant resistance (Table 3). These small changes in resistance were not accompanied by a significant decrease in *NANOG* or *POU5F1* mRNA expression (Figure 7B). Exposure to 10 µM RA did produce an increase of fibronectin mRNA suggesting that GCT27 cells grown in this manner have some small capacity for differentiation in response to RA. Thus, germ cell tumor cell lines that fail to undergo significant differentiation in response to RA or cDDP exposure also fail to acutely develop cDDP resistance.

### Ability of RA and cDDP to Induce Drug Resistance is Dependent upon NANOG Down-Regulation

**Figure 8:**
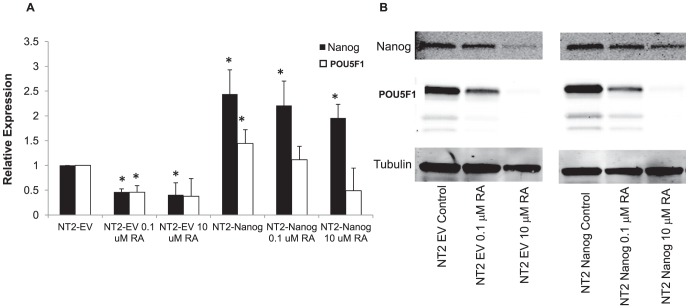
NT2-D1 cells over-expressing NANOG are resistant to RA-induced down-regulation of NANOG and POU5F1. NT2-EV or NT2-NANOG cells were exposed to RA for 2 days prior to examination of NANOG and POU5F1 levels by: A) qRT-PCR; and, B) western blot analysis. qRT-PCR data was normalized to GAPDH. Each bar in the histogram represents the mean of 3 independent experiments; vertical bars, ±SEM. *p<0.05.

In order to determine whether the ability of RA and cDDP to down-regulate the expression of NANOG and POU5F1 is essential to their ability to acutely induce resistance to cDDP, NT2-D1 cells were molecularly engineered to constitutively express *NANOG* by infection with a retroviral expression vector. Control NT2-D1 cells were infected with an empty vector (NT2-EV) which did not alter the expression of NANOG or POU5F1. As shown in Figure 8, in cells infected with a *NANOG*-expressing vector (NT2-NANOG) the *NANOG* mRNA was 2.4-fold higher than in the NT2-EV. Interestingly, over-expression of *NANOG* resulted in a 1.4-fold increase in the level of *POU5F1* mRNA. These two transcription factors are both involved in positive feedback mechanisms, so this finding was not unexpected [24]–[26]. Attempts to create a stable undifferentiated POU5F1 over-expressing cell line were unsuccessful and may be related to the fact that higher POU5F1 over-expression has been reported to induce differentiation [27], [28].

**Figure 9:**
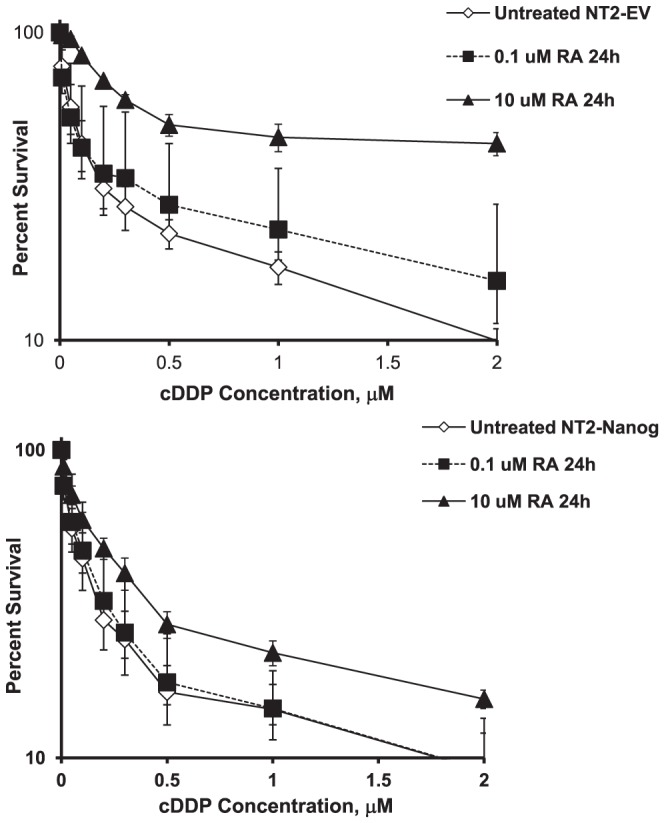
NT2-D1 cells over-expressing NANOG are resistant to induction of cDDP resistance following RA pretreatment. NT2-EV or NT2-NANOG cells were exposed to RA for 1 day then plated and assessed for sensitivity to cDDP 2 days after initiation of treatment by continuous drug exposure using the crystal violet staining assay. Each data point presents the mean of 3 independent experiments each performed with triplicate cultures. Vertical bars, ±SEM.

The NT2-NANOG cells were then tested for their response to pretreatment with RA. For these experiments, NT2-EV or NT2-NANOG cells were exposed to RA for 48 h before analysis. Similar to the parental cells, vector control NT2-EV cells responded to RA with a drop in NANOG and POU5F1 at both the mRNA and protein level (Figure 8). In contrast, an equivalent exposure to RA failed to decrease the level of *NANOG* mRNA in the NT2-NANOG cells to the same amount. *POU5F1* suppression was also attenuated, although at higher RA concentrations *POU5F1* expression could still be suppressed. Consistent with these findings, a 24 h RA pretreatment also failed to induce significant resistance to cDDP in the NT2-NANOG cells. A low level of resistance could be induced with higher RA pretreatment but this was not statistically significant (Figure 9 and Table 4). In contrast, the NT2-EV cells still gained a measurable amount of cDDP resistance with the same treatment which reached statistical significance in the IC_90_ values. It should be noted that in these experiments, shorter durations of RA treatment were required to produce this effect as longer treatments were still capable of inducing differentiation and resistance in the NT2-NANOG cells similar to the parental cells (data not shown).

**Table 4:** IC_50_ and IC_90_ for cDDP in NT2-EV or NT2-NANOG cells pretreated with retinoic acid for 24 hours.

	cDDP IC_50_ µM	Fold Increase	p Value	cDDP IC_90_ µM	Fold Increase	p Value
NT2-EV No pretreatment	0.18±0.01	-		4.3±1.8	-	
NT2-EV pretreated with 0.1 µM RA	0.68±0.23	3.7	NS	5.8±2.4	1.4	NS
NT2-EV pretreated with 10 µ RA	2.03±0.96	11.3	NS	9.4±3.8	2.2	<0.001
NT2-NANOG No pretreatment	0.16±0.01	-		4.9±1.9		
NT2-NANOG pretreated with 0.1 µM RA	0.17±0.01	1.1	NS	3.4±1.4	0.7	NS
NT2-NANOG pretreated with 10 µM RA	0.37±0.06	2.3	NS	6.8±2.8	1.4	NS

## Discussion

Current models of GCT differentiation suggest that embryonal carcinoma and seminoma are the undifferentiated types of tumors and that they share many features with normal embryonic stem cells including expression of pluripotency factors such as NANOG, POU5F1, and SOX-2 among others. The other histologic types of GCTs traditionally include yolk-sac tumors, choriocarcinomas and teratomas. These types of tumors are believed to be derived from the more differentiated cells [1]. Consistent with this premise, these tumors also express lower levels of these pluripotency factors and increased expression of genes associated with more differentiated tissues. That NT2-D1 cells also express NANOG and POU5F1, and can also be induced to differentiate with a subsequent decrease in expression of these factors, supports the hypothesis that these cells represent a true embryonal carcinoma cell line.

The pathways involved in differentiation and apoptosis are tightly regulated in both embryonic stem (ES) cells and primordial germ cells. The results of this study suggest that the linkage between differentiation and resistance to apoptosis found in non-malignant ES and primordial germ cells is maintained when these cells become malignant. This linkage is not commonly found in other tumor types. There is evidence in ES cells and GCTs that mediators of the DNA damage response, such as p53 or the caspases, are involved in differentiation [29]–[32]. Indeed, it is likely that these linkages exist to preserve the integrity of the genome during development and germ cell formation. The results presented here are consistent with the concept that, in embryonal GCTs, the DNA damage response initiated by formation of cDDP DNA adducts leads to induction of both differentiation and apoptotic programs, and that the relative robustness and balance of these programs determines cell fate. Clinical observations in patients with GCTs support this possibility as cDDP or CBDCA-based chemotherapy typically leads to a significant shrinkage of the tumor but persistence of residual masses usually containing tumor of a differentiated histology. Our finding that cDDP triggers differentiation in an embryonal GCT cell line, and that this renders the cells resistant to retreatment with this drug, provides a cogent explanation for this clinical observation.

The observations reported in this work have potentially important clinical implications. The observation that even a single brief exposure to cDDP can induce substantial resistance within several days suggests that the seeds of eventual failure may be laid very early in the treatment process. This suggests that the initial intensity of cDDP therapy, even just the first dose, may be an important determinant of eventual cure. For this tumor in particular, rapid reduction in tumor burden such that the number of surviving cells at risk for differentiation is low may be important. This clinical extrapolation can only be made for non-seminomatous GCTs at this time since we only examined one differentiatable embryonal carcinoma cell line and clearly our findings need to be further validated. While there has been an effort to reduce treatment intensity in seminoma, our results serve to caution clinical investigators who might attempt to apply similar dose reduction strategies in to the management of non-seminomatous GCT treatment.

The role of NANOG and POU5F1 in maintaining pluripotency is well established in ES cells, and there is abundant data supporting their importance in primordial germ cells and GCTs as well. Mueller *et al.* [33] previously reported that loss of POU5F1 expression induced by differentiating GCT cell lines with a medium containing fetal bovine serum and steroids resulted in cDDP resistance, and this has been confirmed by knockdown of POU5F1 [6]. This is consistent with other studies reporting that differentiation of embryonal carcinoma cells leads to induction of resistance [20]. However, NANOG and POU5F1 may play roles in apoptosis as well. It was recently reported that knockdown of NANOG or POU5F1 in mouse ES cells causes apoptosis as well as up-regulation of p53-dependent factors [34], suggesting that these genes also regulate the apoptotic threshold in addition to maintaining pluripotency.

cDDP produced a large decrease in the expression of both NANOG and POU5F1 in the NT2-D1 cells in association with the acute appearance of cDDP and paclitaxel resistance. The same was observed for RA. The finding that cDDP and RA failed to induce acute cDDP resistance in the NT2-D1 cells engineered to over-express *NANOG* indicates that the RA and cDDP-induced reduction in this transcription factor is important in the emergence of the cDDP-resistant phenotype. This is further supported by the finding that RA and cDDP failed to reduce NANOG or POU5F1 expression in the GCT27 and SuSa cells and that this was associated with failure to induce cDDP resistance. The mechanism by which RA and cDDP reduce *NANOG* and *POU5F1* mRNA remains to be defined. Previous studies have suggested that RA can accomplish this by triggering their degradation as well as reducing their mRNA levels. Caspase activation may play a role by virtue of the ability of the caspases to degrade transcription factors such as NANOG and POU5F1 [30]. Musch et al. [29] found that caspase activation was also important for NANOG and POU5F1 degradation in response to cytotoxic nucleosides. We surmise that cDDP induces differentiation utilizing a similar mechanism involving caspase-mediated degradation of NANOG and POU5F1 as caspases are activated subsequent to the formation of Pt-DNA adducts.

It is still unclear whether over-expression of NANOG leads to increased resistance to differentiation through its effects on POU5F1 expression versus other NANOG regulated targets. Unfortunately, attempts to create a subline over-expressing POU5F1 were unsuccessful. However, the data presented hints that the former may be the situation since acquisition of resistance correlated better with loss of POU5F1 rather than NANOG in the NT2-NANOG cells with increasing RA treatment. This would be consistent with the recent findings of Gutekunst et al. [6] who showed that POU5F1 expression is essential to provide the pro-apoptotic environment needed to maintain drug sensitivity in undifferentiated NT2-D1 cells.

The ability of RA and cDDP to acutely induce resistance appears to be unique to a subclass of GCTs in which differentiation can be induced. No induction of acute cDDP resistance could be demonstrated in either the cervical or prostate cancer lines tested. Among the GCTs tested, only those able to undergo differentiation in response to RA or cDDP developed resistance to cDDP. The low level of inducible resistance in the GCT27 cells may be due to the presence of only a minor subpopulation capable of undergoing differentiation as fully differentiatable sublines have been derived from the GCT27 line by others [14], [35]. Mueller *et al.* also reported that H12.5 or 2102EP cells, which could not be differentiated in differentiation-inducing medium, also did not undergo a change in cDDP in sensitivity [33]. These observations further underscore the differences between GCTs and other tumors. In most other types of cancer, the undifferentiated state is associated with a more malignant and chemotherapy-resistant phenotype [36]–[39]. In these other types of cancer higher NANOG and POU5F1 expression has been associated with greater resistance to chemotherapeutic agents and drug resistance has variously been attributed to mesenchymal-like characteristics and increased drug efflux [40]–[42]. GCTs appear to have an opposite relationship between differentiation and drug resistance, where the more undifferentiated histologic types of GCTs such as seminoma are associated with higher chemotherapy sensitivity and better prognosis. Thus, there is something special about the undifferentiated state of GCTs that distinguish them from the undifferentiated stem cells of other tumor types. As mentioned earlier, GCTs are believed to be derived from primordial germ cells which are inherently sensitive to DNA damage and this sensitivity may exist in germ cells to preserve the integrity of the genome during development and germ cell formation [7], [8]. This is a property which may have been inherited by GCTs. However, we note this study is limited by the fact that the NT2-D1 line is the only GCT line we tested which can undergo significant differentiation, and replication of our findings in other differentiable GCT would be supportive of our hypotheses.

In those GCTs that can be differentiated, RA and cDDP acutely induce resistance to both cDDP and paclitaxel. The primary target of cDDP is DNA while microtubules are the primary target of paclitaxel, and these two drugs have very different influx and efflux systems. Thus RA and cDDP must be influencing a final common pathway of cell death rather than a process unique to the cellular pharmacology of one or the other drug. This concept is reinforced by recent studies by Gutekunst *et al.* [43], [44] who found that knockdown of Oct4 resulted in resistance not only to cDDP but also to etoposide and doxorubicin. They found that this was due to reduced expression of the pro-apoptotic proteins Noxa and Puma such that the ability of p53 to trigger apoptosis was reduced. Whether cDDP produces a similar alteration in the balance of pro- and anti-apoptotic proteins will be the subject of future investigation. In contrast, paclitaxel was not able to induce similar changes in NANOG or POU5F1 expression nor induction of resistance to itself or other agents under the conditions tested. Paclitaxel is believed to lead to cell death through mitotic spindle arrest in contrast to cisplatin which induces apoptosis through a DNA damage response. The differing mechanisms of drug action result in complex dependencies on signaling cascades including p53, PI3K, p38, ERK, or JNK/SAPK [45]–[47]. The MAPK pathways in particular have already been shown to control differentiation of embryonic stem cells and notably RA activates these pathways as well [48]. Given our observations and previous studies, one can hypothesize that paclitaxel fails to activate the differentiation pathways triggered by cDDP. Alternatively, paclitaxel treatment may activate signaling pathways which simply do not intersect with the cellular mechanisms influencing differentiation in this cell line. Whatever the reason, downstream events may also involve caspase activation in cDDP or RA induced differentiation as described above [29], [30], [46].

There is now substantial data from genomic studies indicating that resistance acquired during treatment is the result of enrichment for resistant clones already present in the population of tumor cells [49], [50]. It remains possible that the resistance seen in this study was due to clonal selection for pre-existing partly differentiated and resistant clones; however, we think unlikely. If this was the case, one would not expect forced over-expression of NANOG to cause the pre-existing resistant cells to become more sensitive after drug treatment. Previous studies attempting to over-express pluripotency factors in GCTs have not been successful in reversing resistance once differentiation has occurred [33]. cDDP is a good mutagen and we have previously shown that mutagenesis is a mechanism of acquired cDDP resistance. cDDP readily generates new mutant clones that are resistant to cDDP and many of the drugs that are commonly used in combination with cDDP [51]. The data reported in the current paper provide evidence that induction of differentiation is an additional mechanism by which resistance to this important drug is acquired in GCTs.

## Supporting Information

Figure S1 Time course of reduction in *NANOG* and *POU5F1* expression induced by 10 µM RA. qRT-PCR data was normalized to GAPDH. Each bar in the histogram represents the mean of 3 independent experiments; vertical bars, ±SEM.
(EPS)
